# Was Motorized Spiral Enteroscopy Too Risky? A Systematic Review and Meta‐Analysis Including German Registry Data

**DOI:** 10.1002/ueg2.70165

**Published:** 2026-01-06

**Authors:** Ingo Steinbrück, Armin Kuellmer, Siegbert Faiss, Hendrik Buchholz, Björn Lewerenz, Daniel Fitting, Felix Wiedbrauck, Stephan Hollerbach, Arthur Schmidt, Johannes Wilhelm Rey, Martha M. Kirstein, Franz‐Ludwig Dumoulin, Fabian Maximilian Wittich, Andreas Wannhoff, Jürgen Pohl, Matthias Friesicke, Viktor Rempel, Hans‐Peter Allgaier, Christian Wiessner, Thomas Rösch

**Affiliations:** ^1^ Department of Medicine and Gastroenterology Evangelisches Diakoniekrankenhaus Freiburg Academic Teaching Hospital University of Freiburg Freiburg Germany; ^2^ Department of Medicine II Medical Center Faculty of Medicine University of Freiburg Freiburg Germany; ^3^ Department of Gastroenterology Sana Klinikum Lichtenberg Academic Teaching Hospital University of Berlin Berlin Germany; ^4^ Department of Gastroenterology and Hepatology Klinikum Traunstein Academic Teaching Hospital University of Munich Traunstein Germany; ^5^ Department of Gastroenterology Allgemeines Krankenhaus Celle Academic Teaching Hospital University of Hannover Celle Germany; ^6^ Department of Gastroenterology Hepatology and Endocrinology Robert‐Bosch‐Krankenhaus Academic Teaching Hospital University of Tübingen Stuttgart Germany; ^7^ Department of Internal Medicine II Klinikum Osnabrück Academic Teaching Hospital University of Münster Osnabrück Germany; ^8^ Department of Medicine I University Hospital Lübeck University Hospital of Schleswig‐Holstein Lübeck Germany; ^9^ Department of Medicine and Gastroenterology Gemeinschaftskrankenhaus Bonn Academic Teaching Hospital University of Bonn Bonn Germany; ^10^ Department of Gastroenterology RKH Klinikum Ludwigsburg Academic Teaching Hospital University of Heidelberg Ludwigsburg Germany; ^11^ Department of Gastroenterology Asklepios Klinik Altona Academic Teaching Hospital University of Hamburg Hamburg Germany; ^12^ Department of Gastroenterology St. Anna Hospital Herne Academic Teaching Hospital Ruhr University Bochum Bochum Germany; ^13^ Institute for Medical Biometry and Epidemiology University Hospital Hamburg‐Eppendorf Hamburg Germany; ^14^ Department of Interdisciplinary Endoscopy University Hospital Hamburg‐Eppendorf Hamburg Germany

**Keywords:** balloon enteroscopy, device‐assisted enteroscopy, enteroscopy, motorized spiral enteroscopy, small bowel endoscopy

## Abstract

**Background and Study Aims:**

Motorized spiral enteroscopy (MSE) was introduced as a major advancement in small‐bowel enteroscopy, enabling higher complete enteroscopy rates with shorter procedure times. However, after a fatal adverse event (AE) involving severe esophageal injury, the device was withdrawn from the market in July 2023. This raised questions about whether earlier safety signals were missed.

**Methods:**

We conducted a systematic review and meta‐analysis comparing MSE with balloon‐based enteroscopy (double‐balloon [DBE] and single‐balloon enteroscopy [SBE], analyzed together). Outcomes included overall AEs, serious AEs (SAEs), and data collection quality. Results from the German PowerSpiral Registry were included, comprising 647 MSE procedures in 523 patients (January 2020–July 2023) before registry closure following device withdrawal.

**Results:**

Thirteen MSE studies (including the registry) and 55 DBE/SBE studies were analyzed, totaling 12,559 enteroscopies (2024 MSE; 10,535 DBE/SBE). MSE showed significantly higher rates of AEs (10.8% vs. 1.6%) and SAEs (1.5% vs. 0.4%). Procedure‐related SAEs were also more frequent with MSE (1.1% vs. 0.3%). Esophageal injury (0.10% vs. 0.009%) and intestinal perforation (0.5% vs. 0.1%) occurred more often with MSE, whereas acute pancreatitis (0.05% vs. 0.27%) and esophageal perforation (0% vs. 0.02%) were more common with DBE/SBE. AE reporting for MSE was detailed, but structured follow‐up and reliable case tracking were inconsistent.

**Conclusions:**

MSE was associated with higher AE and SAE rates than balloon enteroscopy. These findings highlight the need for cautious adoption, rigorous safety monitoring, and more robust AE reporting when introducing innovative endoscopic technologies.

**Study Registration:**

The prospective and retrospective cohort studies were registered in the German Registry of Clinical Studies (DRKS), namely DRKS00026990 and DRKS00028571

## Summary

- Summarize the established knowledge on this subject.
  ◦ Motorized PowerSpiral Enteroscopy (MSE) demonstrated shorter procedure times, deeper intestinal access, and higher complete enteroscopy rates than earlier enteroscopy techniques.
  ◦ Early safety studies reported AE rates within European guideline thresholds, suggesting that MSE was safe in clinical use.
  ◦ Despite these promising outcomes, the device was withdrawn from the market in July 2023 following safety concerns after a fatal esophageal perforation.
- What are the significant and/or new findings of this study?
  ◦ MSE was associated with higher adverse event rates, including serious events, compared with balloon enteroscopy.
  ◦ Cautious use and strengthened adverse event monitoring are essential when introducing new and innovative endoscopic technologies.

## 1 Introduction

Device‐assisted enteroscopy techniques—such as double‐balloon enteroscopy (DBE), single‐balloon enteroscopy (SBE), and spiral overtube systems—are well‐established for small bowel evaluation but are limited by long procedure times and low complete enteroscopy rates [1, 2, 3]. In 2017, motorized spiral enteroscopy (MSE; PowerSpiral, Olympus Medical Systems, Tokyo, Japan) was introduced, showing promise with shorter procedure times, deeper insertion, and higher complete enteroscopy rates than previous methods [4, 5, 6, 7]. Initial safety studies reported serious adverse event (SAE) rates below the 5% threshold defined by the ESGE [8, 9], while adverse event (AE) rates varied across three comparative studies with DBE/SBE [10, 11, 12]. However, in July 2023, MSE device was withdrawn from the market due to safety concerns after a fatal case of esophageal perforation occurred when the spiral overtube became lodged in the upper esophagus [13].

Although the incident was tragic, the global withdrawal of MSE sparked dissatisfaction among many users, as the system had been viewed as a major advancement in small bowel endoscopy. This has raised debate over whether the withdrawal was justified, given the previously promising safety data reported in the literature. Addressing this question requires a critical appraisal of all available evidence. To this end, we conducted a systematic review and meta‐analysis comparing the safety of MSE with DBE/SBE, incorporating data from the largest multicenter registry study on MSE to date, which was terminated following the device's withdrawal. This analysis aimed to determine whether safety concerns were previously overlooked and if earlier recognition by the endoscopic community could have altered the course of adoption.

## 2 Methods

### 2.1 Definitions and Outcomes

To ensure consistent classification of AE and SAE in the systematic review and registry analysis, events were defined or reclassified when appropriate according to the American Society for Gastrointestinal Endoscopy (ASGE) lexicon and the AGREE classification system [14].

#### 2.1.1 Primary Outcomes

- AE: Any adverse, unintended event that interrupts completion of the planned procedure and/or leads to hospitalization, prolonged hospital stay, additional procedures requiring sedation/anesthesia, or subsequent medical consultation.
- SAE: A severe AE resulting in death or a potentially life‐threatening condition, corresponding to a moderate/severe/fatal AE in the ASGE lexicon or AGREE grade II–V [14].

#### 2.1.2 Secondary Outcomes

Procedure‐/device‐related AEs and SAEs (=not related to therapeutic interventions) for the meta‐analysis, for secondary outcomes in the registry study Table S1.

### 2.2 Systematic Review and Meta‐Analysis

The review followed PRISMA guidelines. Eligible studies included clinical trials, cohort studies, comparative studies, randomized controlled trials, and case series with more than 20 patients. Only MSE or DBE/SBE procedures for small‐bowel indications with available AE and SAE data were included. Studies were excluded if they involved non‐small bowel procedures (e.g., ERCP, colonoscopy) or highly selective cohorts (e.g., Crohn's disease, Peutz–Jeghers syndrome, and pediatric populations). We did not include reviews, abstracts, conference reports, incomplete data, or non‐English publications either.

#### 2.2.1 Search Strategy

The initial search was conducted in the PubMed/MEDLINE, Cochrane, and ClinicalTrials.gov databases on December 12, 2024. Boolean search terms were applied to identify publications from January 1, 2001, to December 12, 2024. The search terms included: “motorized spiral enteroscopy,” “motorized spiral endoscopy,” “novel spiral enteroscope,” “novel spiral endoscope,” “spiral endoscopy,” “power spiral,” “double balloon endoscopy,” “double‐balloon endoscopy,” “single balloon endoscopy,” “single‐balloon endoscopy,” “device‐assisted endoscopy,” “push‐and‐pull endoscopy,” “balloon‐assisted endoscopy,” “double balloon enteroscopy,” “double‐balloon enteroscopy,” “single balloon enteroscopy,” “single‐balloon enteroscopy,” “device‐assisted enteroscopy,” “push‐and‐pull enteroscopy,” and “balloon‐assisted enteroscopy.”

Additional relevant articles were identified through reference lists of retrieved studies and the “Similar Articles” feature in PubMed/MEDLINE. Two independent reviewers (I.S. and A.K.) selected, documented, and standardized the articles based on predefined inclusion and exclusion criteria. In cases of duplicate data or multiple publications from the same study, only the most recent and complete version was included. Discrepancies were resolved by consensus with a third reviewer (H.P.A.).

In addition, extracted data included study design, number of procedures, case inclusion monitoring, adequacy of follow‐up (minimum 7 days), AE assessment methods, and overall methodological quality. Study quality was evaluated using the modified Newcastle–Ottawa Scale (NOS), adapted for cohort studies without a comparator [15, 16]. We also conducted a targeted review of MSE safety, focusing on esophageal AEs and device failures, summarizing all published evidence, and including case reports and case series not eligible for the meta‐analysis.

#### 2.2.2 Statistical Analysis

A generalized linear mixed model with a logit transformation was used for pooled proportions and 95%‐confidence intervals (CI). Overall and between study heterogeneity was measured using τ^2^ and the inconsistency index (I^2^) with cutoffs for heterogeneity of 25% (low), 50% (moderate), and 75% (high) [17]. The pooled proportions for AEs and SAEs were calculated per procedure and separately for MSE and DBE/SBE trials. The analyses were carried out with the R package meta (Version 7.0–0).

### 2.3 Registry Study

The registry data used was an investigator‐initiated, multicenter observational cohort study, combining data from both the prospective and retrospective German PowerSpiral (GPS) registries from 11 German centers. The registries were initiated after approval by the responsible ethics committee of the University of Freiburg and the local ethics committees of the recruiting hospitals. The study was conducted in accordance with the ethical guidelines of the Declaration of Helsinki, and no funding was received. Some of the cases included in the registry study have previously been published as case reports/series in different contexts [7, 18, 19]. Further study details are provided in the Table S2. Analyses of AE and SAE were performed for the overall cohort (all indications) and in the subgroups small bowel, ERCP, and colonoscopy. The cases with small bowel indication were also included in the meta‐analysis. A doubling of data was avoided because the previously published case reports/series [7, 18, 19] did not meet the inclusion criteria for the meta‐analysis. Procedures were performed using the PSF‐1 PowerSpiral enteroscope (Olympus Medical Systems Corporation, Tokyo, Japan) by experienced endoscopists trained in device‐assisted enteroscopy and certified through the manufacturer's hands‐on training program, a prerequisite for performing MSE. Interventions followed the company's standardized protocol [4, 6] and were carried out via peroral and/or peranal routes, as determined by the examiner based on prior diagnostic findings.

#### 2.3.1 Statistical Analysis

To identify independent predictors for the primary endpoints, a univariable logistic regression model was used, followed by a multivariable logistic regression model including those factors that were associated with the dependent outcomes in the univariable analysis (*p*‐value < 0.1). In multivariable analysis, the backward selection method was performed with a significance level of *p* < 0.05.

## 3 Results

### 3.1 Systematic Review and Meta‐Analysis

**FIGURE 1:**
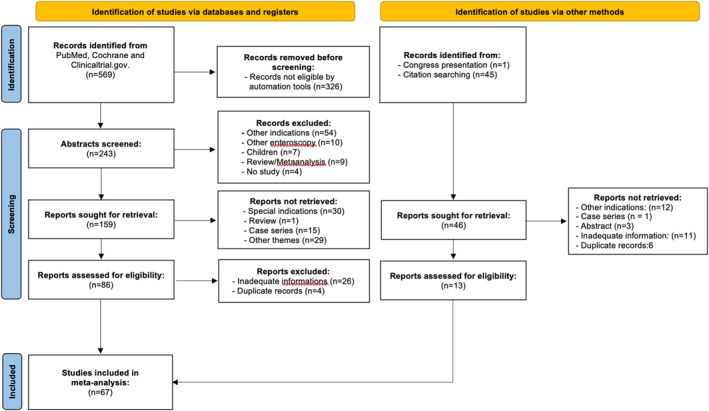
Flow sheet of the meta‐analysis (with inclusion of publications from January 1 2001, to December 12 2024).

A total of 615 studies were identified through the search strategy. Of these, 326 were excluded by automated tools due to ineligibility. After screening 289 abstracts, 222 full‐text articles were assessed, and 67 studies met the inclusion criteria for meta‐analysis. Including our own data, 13 studies reported data on MSE (publication years 2020–2025) [4, 5, 6, 8, 10, 12, 20, 21, 22, 23, 24, 25, own study], and 55 on DBE/SBE (2005–2024) [2, 12, 26, 27, 28, 29, 30, 31, 32, 33, 34, 35, 36, 37, 38, 39, 40, 41, 42, 43, 44, 45, 46, 47, 48, 49, 50, 51, 52, 53, 54, 55, 56, 57, 58, 59, 60, 61, 62, 63, 64, 65, 66, 67, 68, 69, 70, 71, 72, 73, 74, 75, 76, 77, 78] with one RCT that included both techniques [12] (Figure 1). Among the included studies, prospective design was present in 53.8% of MSE and 45.5% of DBE/SBE studies; multicenter studies accounted for 46.2% of MSE and 21.8% of DBE/SBE; and RCTs were found in 7.7% and 20.0%, respectively. Inclusion monitoring was reported in 23.1% (MSE) and 20.0% (DBE/SBE), while adequate follow‐up (≥ 7 days) was documented in 53.8% of MSE and 20.0% of DBE/SBE studies (Table 1). An overview of the included studies is provided in Table S3.

**TABLE 1:** Characteristics of studies included in the meta‐analysis (small bowel indications).

	MSE‐studies (*n* = 13)	DBE/SBE‐studies (*n* = 55)
Prospective	7 (53.8%)	25 (45.5%)
Multicentric	6 (46.2%)	12 (21.8%)
RCT	1 (7.7%)	11 (20.0%)
Monitoring of inclusion	3 (23.1%)	11 (20.0%)
FU (minimum of 7 days)	7 (53.8%)	11 (20.0%)
NOS		
Poor	1 (7.7%)	27 (49.1%)
Moderate	5 (38.5%)	24 (43.6%)
Good	7 (53.8%)	4 (7.3%)
Objectifiable definition of AE	7 (53.8%)	4 (7.3%)

Abbreviations: AE: Adverse event, DBE: Double‐balloon enteroscopy, FU: Follow‐up, MSE: Motorized spiral enteroscopy, NOS: Newcastle‐Ottawa Scale, RCT: Randomized controlled trial, SBE: Single‐balloon enteroscopy.

Quality assessment using the modified NOS [15, 16] rated MSE studies as poor in 7.7%, moderate in 38.5%, and good in 53.8%. In contrast, DBE/SBE studies were rated poor in 49.1%, moderate in 43.6%, and good in only 7.3%. Two MSE (15.4%) and two DBE/SBE studies (3.6%) had a primary focus on safety [7,40,66, own study]. Definitions and reporting of AEs were highly heterogeneous. An objective AE scoring system was applied in 53.8% of MSE studies but only 7.3% of DBE/SBE studies.

**FIGURE 2:**
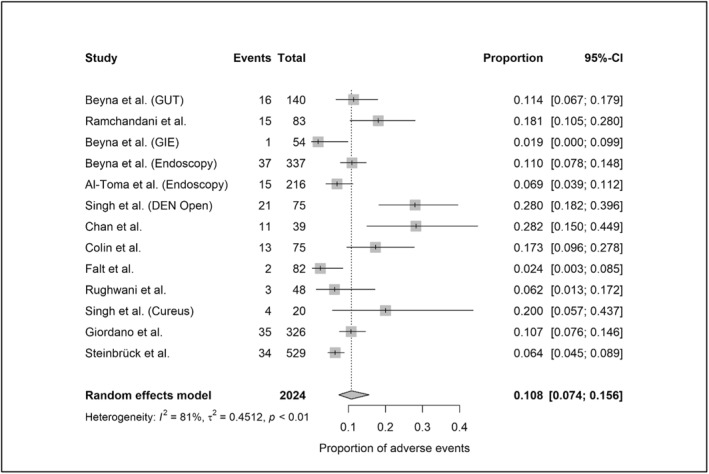
AE in MSE (small bowel indications)—Forest plot of the meta‐analysis.

**FIGURE 3:**
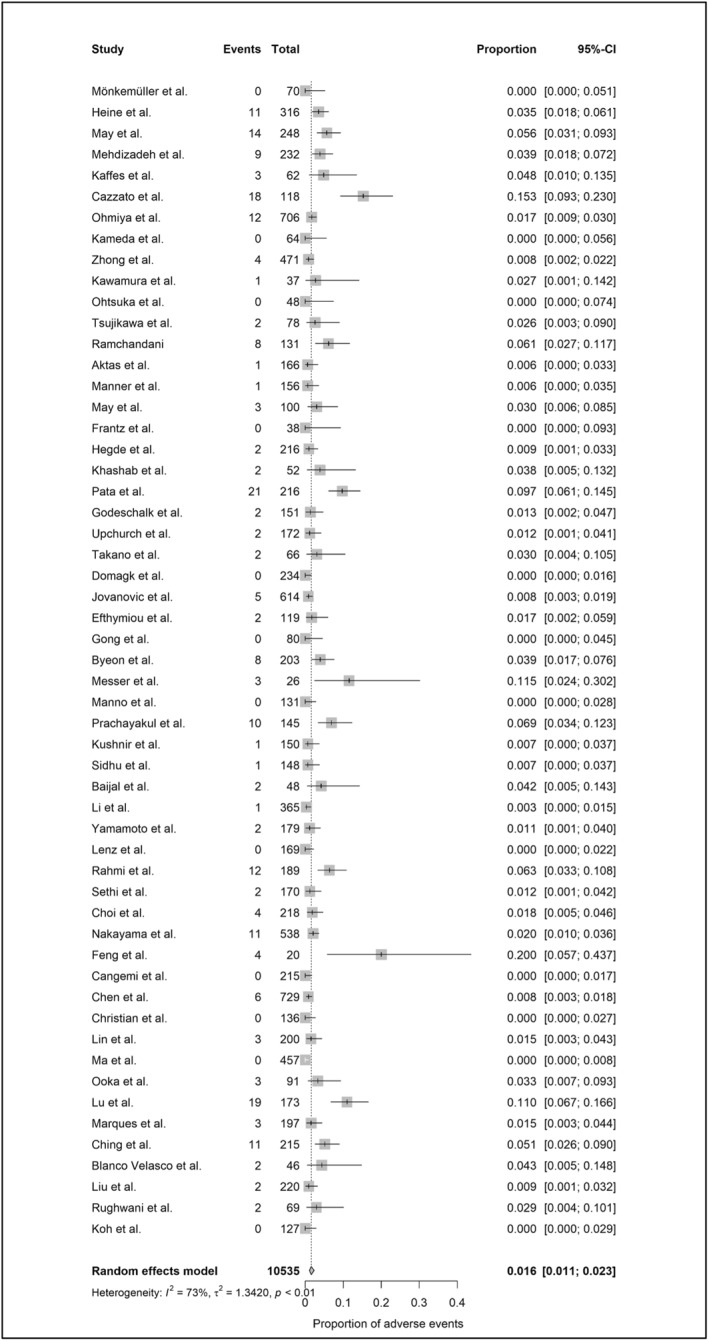
AE in DBE/SBE (small bowel indications)—Forest plot of the meta‐analysis.

**FIGURE 4:**
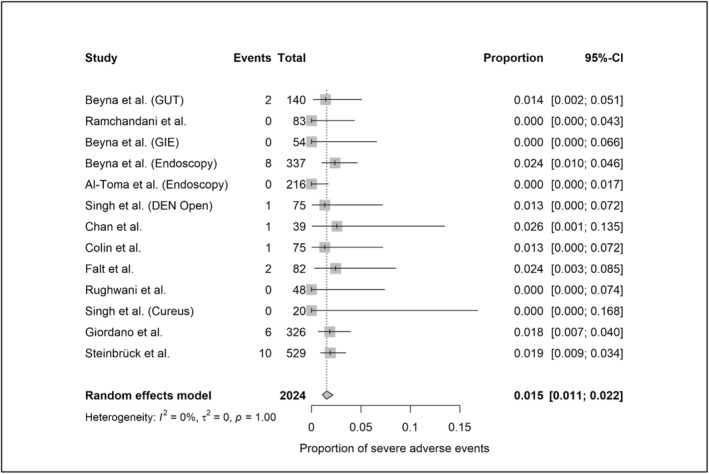
SAE in MSE (small bowel indications)—Forest plot of the meta‐analysis.

**FIGURE 5:**
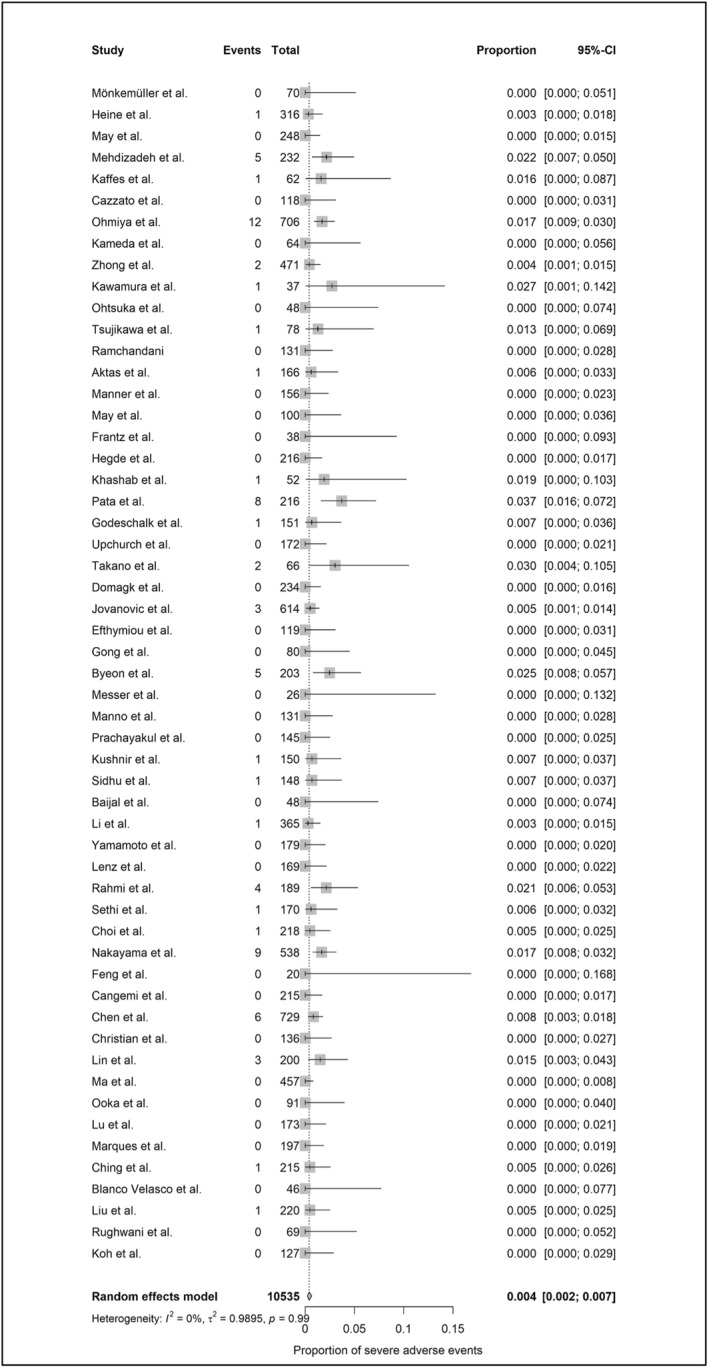
SAE in DBE/SBE (small bowel indications)—Forest plot of the meta‐analysis.

A total of 12,559 device‐assisted enteroscopy procedures were included in the meta‐analysis (2024 MSE and 10,535 DBE/SBE). Per‐procedural AE rates, based on standardized definitions, were 10.8% (95%‐CI: 7.4%–15.6%; heterogeneity: 81%) for MSE (Figure 2) and 1.6% (95%‐CI: 1.1%–2.3%; heterogeneity: 73%) for DBE/SBE (Figure 3). SAE rates were 1.5% (95%‐CI: 1.1%–2.2%; heterogeneity: 0%) for MSE (Figure 4) and 0.4% (95%‐CI: 0.2%–0.7%; heterogeneity: 0%) for DBE/SBE (Figure 5). Procedure‐related AEs occurred in 9.5% (95%‐CI: 6.1%–14.3%; heterogeneity: 82%) for MSE and 1.3% (95%‐CI: 0.8%–2.0%; heterogeneity: 73%) for DBE/SBE. Procedure‐related SAE rates were 1.1% (95%‐CI: 0.7%–1.6%; heterogeneity: 0%) for MSE and 0.3% (95%‐CI: 0.1%–0.5%; heterogeneity: 0%) for DBE/SBE.

A total of 31 SAEs were reported across eight MSE studies, encompassing 2024 procedures [6,8,10,22–24,26, own study]. The most common events were intestinal perforations (35.5%), difficulties during device withdrawal (16.1%), and sedation‐related cardiopulmonary complications (12.9%) (Table 2). Of the 11 perforations, eight were managed surgically, two endoscopically, and one conservatively. All SAEs were successfully resolved, with no reported mortality.

**TABLE 2:** SAE in MSE‐ and DBE/SBE‐studies included in the meta‐analysis (small bowel indications).

	SAE in MSE (31/2024)	SAE in DBE/SBE (73/10,535)
Acute pancreatitis	1 (3.2%)	28 (38.4%)
Bleeding	3 (9.7%)	7 (9.6%)
Intestinal perforation	11 (35.5%)	15 (20.5%)
Esophageal perforation	0	2 (2.7%)
Intestinal mucosal injuries	3 (9.7%)	2 (2.7%)
Esophageal mucosal injuries	2 (6.5%)	1 (1.4%)
Intestinal ischemia	1 (3.2%)	0
Paralytic ileus	0	2 (2.7%)
Withdrawal problems	5 (16.1%)	0
Defect of the device	1 (3.2%)	0
Sedation related cardial or pulmonary SAE	4 (12.9%)	15 (20.5%)
Sepsis	0	1 (1.4%)

Abbreviations: DBE: Double‐balloon enteroscopy, MSE: Motorized spiral enteroscopy, SAE: Serious adverse event, SBE: Single‐balloon enteroscopy.

In the DBE/SBE group, 73 SAEs were reported across 25 studies involving 10,535 procedures [28, 29, 30, 32, 34, 35, 37, 39, 44, 45, 46, 48, 50, 53, 56, 57, 59, 62, 63, 64, 65, 68, 70, 75, 77]. The most common complications were acute pancreatitis (38.4%), intestinal perforation (20.5%), and sedation‐related cardiopulmonary events (20.5%) (Table 2); also, two esophageal perforations were documented [32, 63]. All 28 cases of pancreatitis were managed conservatively. Of the 15 intestinal perforations, eight were treated surgically and three conservatively. Treatment details were not reported in four cases. One patient died of cardiac arrest within 24 h post‐DBE, possibly related to periprocedural sedation (mortality rate: 0.008%).

The subgroups of peroral and peranal procedures were also analyzed for AEs and SAEs. Although relevant data were lacking in many studies, particularly with regard to AEs, the pooled analysis showed a trend toward more AEs in peroral compared to peranal MSEs (6.1% [95%‐CI: 3.1%–11.8%] vs. 3.2% [95%‐CI: 1.6%–6.2%] but similar rates of SAE (1.7% [95%‐CI: 1.1%–2.5%] vs. 1.3% [95%‐CI: 0.6%–2.6%]). For DBE/SBE, no relevant differences were apparent for AE and SAE with both approaches (0.6% [95%‐CI: 0.2%–2.0%] vs. 0.7% [95%‐CI: 0.2%–2.6%] and 0.1% [95%‐CI: 0.0%–0.5%] vs. 0.1% [95%‐CI: 0.0%–0.9%]). When comparing MSE and DBE/SBE, the AEs and SAEs were higher in the MSE group for both approaches, but only for peroral procedures with no overlap of the 95%‐CI (Table 3, Figure [Link], [Link], [Link], [Link], [Link], [Link], [Link], [Link]).

**TABLE 3:** Subgroup analysis of AE and SAE in peroral and peranal MSE and DBE/SBE (small bowel indications).

	Motorized spiral enteroscopy		Double‐/single‐balloon enteroscopy	
	Peroral	Peranal	Peroral	Peranal
AE	6.1%^a^ [95%‐CI 3.1–0.11.8%]	3.2%^b^ [95%‐CI 1.6%–6.2%]	0.6%^c^ [95%‐CI 0.2%–2.0%]	0.7%^d^ [95%‐CI 0.2%–2.6%]
SAE	1.7%^e^ [95%‐CI 1.1%–2.5%]	1.3%^f^ [95%‐CI 0.6%–2.6%]	0.1%^g^ [95%‐CI 0%–0.5%]	0.1%^h^ [95%‐CI 0%–0.9%]

Abbreviations: 95% CI: 95% confidence interval, AE: Adverse event, SAE: Serious adverse event.

^a^ Pooled analysis from 7 studies (*n* = 697).

^b^ Pooled analysis from 5 studies (*n* = 251).

^c^ Pooled analysis from 21 studies (*n* = 1636).

^d^ Pooled analysis from 19 studies (*n* = 1213).

^e^ Pooled analysis from 12 studies (*n* = 1391).

^f^ Pooled analysis from 10 studies (*n* = 558).

^g^ Pooled analysis from 35 studies (*n* = 2978).

^h^ Pooled analysis from 32 studies (*n* = 2179).

Additional SAEs related to MSE reported in case studies not included in the meta‐analysis were two cases of procedure‐induced and one case of ERCP‐induced acute pancreatitis [79, 80], two small bowel perforations [22, 81], one esophageal perforation [80], one biliary leak following ERCP [22], and one case of papillary bleeding [82].

As the device was withdrawn from the market due to a fatal esophageal complication, we specifically analyzed esophageal AEs. Passage‐related issues with MSE were common (Table 4), including numerous clinically relevant deep esophageal lacerations [4, 5, 11, 12, 20, 21, 24, 79, 80], two perforations [80, 83], and nine published cases of overtube entrapment [10, 13, 25, 84, 85]. All overtubes were successfully retrieved without lasting harm, except for the single fatal case that led to market withdrawal [13]. Notably, all these cases were reported after 2020. In comparison, three esophageal perforations were reported with DBE/SBE [32, 63, 83], but clinically relevant esophageal lacerations appeared to be rare [12, 30].

**TABLE 4:** Published cases of esophageal incidents/AE and technical problems with the MSE (studies from the meta‐analysis and other case series/reports including all indications).

Author/year	Type of study	*n*	Indication	Management	Outcome
Esophageal deep lacerations with clinical complaints					
Al‐Toma et al. 2022 [5]	Observational	8/216	Enteroscopy	Observation	Resolved
Beyna et al. 2020 [4]	Observational	1/54	Enteroscopy	Endoscopic clipping	Resolved
Prasad et al. 2020 [79]	Case series	3/14	Enteroscopy	Observation	Resolved
Singh et al. 2022 [21]	Observational	17/75	Enteroscopy	Observation	Resolved
Rughwani et al. 2023 [12]	RCT	3/48	Enteroscopy	Observation	Resolved
Ramchandani et al. 2021 [20]	Observational	15/83	Enteroscopy	Observation	Resolved
Pal et al. 2023 [11]	RCT	1/70	Enteroscopy	Observation	Resolved
Singh et al. 2024 [24]	Observational	2/20	Enteroscopy	Observation	Resolved
Moreels et al. 2024 [80]	Observational	3/88	ERCP	Observation	Resolved
Esophageal perforation					
Shiha et al. 2024 [83]	Observational	1/1	n.s.	n.s.	n.s.
Moreels et al. 2024 [80]	Observational	1/88	ERCP	Endosponge/surgery	Resolved
Spiral stuck in the upper esophagus					
Olympus 2023 [13]	Urgent field safety notice	1	n.s.	Surgery	Death
Giordano et al. 2024 [25]	Observational	4/326	Enteroscopy	Forward/backward maneuver	Resolved
Chan et al. 2023 [10]	Case‐matched	2/39	Enteroscopy	Forward/backward maneuver	Esophageal lacerations
Pal et al. 2023 [84]	Comparative study	1/55	Enteroscopy	Forward/backward maneuver	Resolved
Dray et al. 2024 [85]	Case report	1	Enteroscopy	Manual extraction	Esophageal lacerations
Disconnection of the spiral in the upper esophagus					
Beyna et al. 2022 [8]	Observational	1/337	Enteroscopy	Manual extraction	n.s.
Steiner et al. 2022 [86]	Case report	1	ERCP	Extraction with dilation balloon	Esophageal lacerations
Munoz‐Perez et al. 2022 [87]	Case report	1	Enteroscopy	Extraction with dilation balloon and magill forceps	Esophageal lacerations
Wiedbrauck et al.^a^ 2024 [19]	Case report	1	Enteroscopy	Laryngoscopic extraction meniscus forceps)	Esophageal lacerations
Defect/Detachment of the spiral sheath					
Rajput et al. 2022 [88]	Case report	2	Enteroscopy	Detachment of the fin in the stomach and in the small bowel	Forceps extraction (stomach), spontaneous passage
Barthet et al. 2023 [89]	Case report	1	Enteroscopy	Detachment of the frontal ring of the overtube in the small bowel	Polyethylene glycol to encourage spontaneous passage
Dray et al. 2024 [85]	Case report	1	Enteroscopy	Detachment of the whole sheath in the esophagus	Manual extraction

Abbreviations: AE: Adverse event, ERCP: Endoscopic retrograde cholangiopancreaticography, MSE: Motorized spiral enteroscopy, n.s.: not specified, RCT: randomized controlled study.

^a^ Case also included in the GPS study.

Another important aspect of safe clinical use is the technical reliability of the system. Between 2022 and 2024, four MSE cases were reported in which the spiral overtube detached from the endoscope and required manual or laryngoscopic removal [8, 19, 86, 87], as well as four additional cases involving overtube disintegration [85, 88, 89] (Table 4). In contrast, no comparable technical failures were reported for the SBE/DBE devices.

### 3.2 Registry Study (Overview)

Between 29 January 2020 and 7 July 2023, 647 procedures were performed in 523 patients from 11 participating centers (Table [Link], [Link], [Link], [Link], [Link], [Link], [Link], [Link]) including 147 procedures (123 patients) from the prospective and 500 MSE (400 patients) from the retrospective database (Figure [Link], [Link], [Link], [Link], [Link], [Link], [Link], [Link]). For patient and procedure characteristics, see Table [Link], [Link], [Link], [Link], [Link], [Link], [Link], [Link] 39.2% of patients had previous abdominal surgery. Procedures were successful in 577 cases (89.2%). The most common reasons for unsuccessful procedures were inability to advance the scope in the small bowel (27.1%) or esophagus (11.4%). For further indication‐specific details (see Table [Link], [Link], [Link]).

The AE rate for all indications was 6.2% (40/647; 95% CI: 4.3%–8.2%). Types of AE were intraprocedural bleeding (1.4%), perforation (1.1%), respiratory failure/aspiration (1.2%), acute pancreatitis (0.2%), abdominal pain (0.8%), nasal bleeding (0.3%), fever (0.2%), detachment of the spiral overtube (0.3%) and the overtube stuck in the upper esophagus during withdrawal (0.8%). SAEs occurred in 1.7% of the procedures (seven perforations, one ileal edema, one acute post‐ERCP‐pancreatitis, one aspiration and one detachment of the spiral in the upper esophagus). Surgery was necessary in six cases. Mortality was 0%. Seven cases of technical problems/failures of the MSE device occurred in the upper esophagus (Tables [Link], [Link], [Link]). For predictive factors for AEs, see Table [Link], [Link], [Link].

## 4 Discussion

This paper addresses a critical question: How valid was the assessment of AEs for a new technology such as MSE during its early clinical use and in the published literature? MSE was withdrawn from the market after a fatal esophageal complication. Was this an isolated incident, or could warning signs have been recognized earlier—especially by the endoscopic community? To elucidate this issue, we conducted a systematic literature review and meta‐analysis, including data from a prematurely discontinued German registry that collected MSE cases, both retrospectively and prospectively. Analysis of 12,559 enteroscopies (2024 MSE; 10,535 DBE/SBE) showed that MSE had higher rates of AEs (10.8% vs. 1.6%), SAEs (1.5% vs. 0.4%), and procedure‐related SAEs (1.1% vs. 0.3%), as well as more esophageal injuries (0.10% vs. 0.009%) and intestinal perforations (0.5% vs. 0.1%).

New endoscopic devices require rigorous assessment, especially for safety, which needs large case numbers. While manufacturers hold primary responsibility, MSE appeared to be introduced responsibly: mandatory training and company support were required for early use, and initial studies reported no fatal events. Therefore, the 2023 recall—shortly after a positive safety evaluation analysis [8]—was unexpected within the endoscopic community.

Responsibility also lies with endoscopists and the scientific community. New technologies are often adopted quickly and labeled “safe” based on small retrospective studies, where safety is a secondary outcome. RCTs are rare and usually underpowered to detect uncommon complications. For MSE, only one randomized trial exists (plus one in Crohn's disease) [11, 12], yet three meta‐analyses already concluded it was safe: Nabi et al. (“MSE is a safe and effective tool”) [90], Wang et al. (“MSE… can achieve high diagnostic and therapeutic yields, and relatively low rates of severe adverse events”) [91], and Papaefthymiou et al. (“MSE provides high rates of diagnostic and therapeutic success with a low prevalence of severe adverse events”) [92]. However, early data hinted at risk—for example, a perforation within the first 140 cases [6] and relatively frequent esophageal passage issues as in our series and frequent esophageal passage difficulties in our series. Should these concerns have been recognized sooner? And what role should repeated meta‐analyses play in early safety assessment?

Although MSE has demonstrated excellent effectiveness—also confirmed by our registry—published data on AEs are inconsistent, with reported rates ranging from 10% to 48% [4, 5, 6, 8, 20, 21, 22, 80]. Another key issue is the use of standardized AE classification systems in studies and meta‐analyses. Among the 13 MSE studies included in this review, only seven applied an established classification system [8, 10, 12, 23, 24, and 26, own study]. Although this is better than in DBE/SBE studies—where only four out of 55 used standardized definitions [12, 39, 48, 78]—it remains inadequate. Objective, consensus‐based definitions are essential for accurate AE assessment and to avoid misleading conclusions. For example, under the ASGE AE lexicon and the AGREE classification, intraprocedural events that do not cause symptoms, require treatment, prolong observation, or lead to aborted procedures should not be classified as AEs [14, 93].

Frequent minor or novel complications may signal increased risk for more serious AEs. This is relevant for mucosal injuries, which are common in MSE and often reported as AEs [4, 5, 6, 8, 10, 11, 12, 20, 21, 22]. Although their significance was initially uncertain, their inclusion has driven higher AE rates in recent analyses—17% in meta‐analyses [91, 92] and 21% versus 4.8% [11] and 25.6% versus 1.2% [10] in comparative studies (MSE vs. DBE/SBE). Yet, these meta‐analyses still characterized MSE as safe [90, 91, 92]. Should persistently elevated minor AE rates not prompt greater caution?

In the only RCT comparing MSE and SBE (72 cases), AGREE‐based classification showed similar AE rates (8.5% vs. 5.4%, *p* = 0.66) [12], though numbers were small. In our registry, including asymptomatic mucosal injuries would have increased the AE rate to 26.9%, whereas applying standardized, clinically meaningful criteria resulted in a rate of 6.2%. When we reclassified studies in the meta‐analysis accordingly, the pooled AE rate for MSE was 10.8% (95% CI: 7.4%–15.6%). Although AE rates in DBE/SBE studies may be underestimated due to inconsistent reporting, the pooled rate remained significantly lower at 1.6% (95% CI: 1.1%–2.3%).

Severe AEs are crucial for assessing MSE safety. ESGE guidelines define acceptable SAE rates as 1% for diagnostic and 5% for therapeutic procedures [9]. The two largest prospective studies reported rates of 2.3% and 1.8% [8, 25], while many others reported none [4, 5, 11, 12, 20, 22, 91]. In our cohort, SAEs occurred in 1.7% (1.9% for small bowel cases) with no mortality. The pooled SAE rate in our meta‐analysis was 1.5% (95% CI: 1.1%–2.2%), significantly higher than 0.4% (95% CI: 0.2%–0.7%) for DBE/SBE. Standardized re‐analysis confirmed procedure‐related AE/SAE rates of 9.5%/1.1% for MSE versus 1.3%/0.3% for DBE/SBE. Notably, 75% of SAEs in our registry and 71% in the meta‐analysis were device‐related, mainly perforations.

The influence of operator experience is unclear. In our registry, lower procedural competence was associated with more AEs, whereas age, sex, ASA status, and prior abdominal surgery were not predictive. Whether SAEs decrease with experience remains speculative, though dedicated training is essential. Safety issues may not be limited to peroral MSE: subgroup analysis showed a trend toward more AEs perorally, but AE/SAE rates were also higher than DBE/SBE for peranal procedures (3.2% vs. 0.7% and 1.3% vs. 0.1%). In the registry, approach (pro/retrograde) did not predict AEs. Overall, MSE appears more traumatic than DBE/SBE. Despite strong clinical performance, safety has rarely been the primary focus of published studies—including our own registry initially.

The recall was driven not by overall SAE rates but by a fatal esophageal overtube entrapment [13], making esophageal complications a key safety concern. Beyond five cases in our cohort, nine additional events have been reported [10, 13, 25, 84, 85], along with multiple deep lacerations [4, 5, 11, 12, 20, 21, 24, 79, 80] and two perforations [80, 83]. Failed esophageal access was also frequently noted [6, 20, 21, 23, 24, 94, 95]. The upper esophageal sphincter—the narrowest, most vulnerable point—poses a challenge with MSE due to the overtube's large diameter and rotation. Balloon enteroscopy can also cause esophageal injury but is typically milder and less often. Thus, earlier and more critical discussion of this risk by the endoscopic community would have been appropriate.

Our analysis has limitations. Although the systematic review, including data from a large German multicenter registry, provides a realistic safety evaluation of MSE, many included studies were retrospective, introducing potential selection bias and underreporting due to limited follow‐up. The low number of SAEs restricts statistical power. Differences in sample size, study periods, and AE documentation further reduce comparability between MSE and DBE/SBE studies. Importantly, the generally lower quality and less systematic AE reporting in older DBE/SBE studies may have biased results against MSE by underestimating balloon‐enteroscopy complications.

In conclusion, although MSE meets ESGE safety thresholds, it is more invasive and carries a considerably higher complication risk than DBE/SBE. Its motorized propulsion, limited tactile feedback, and larger diameter necessitate device refinements and dedicated training—especially to ensure safe esophageal passage. We advocate a selective, stepwise approach: MSE for complex or therapeutic cases, and balloon enteroscopy for routine diagnostics or in older patients. Strong safety oversight and transparent reporting from manufacturers and users are essential when introducing new technologies. Post‐marketing studies with complete case inclusion should be mandatory to detect safety issues early, reflecting the scientific community's responsibility. Overall, future research—particularly meta‐analyses—should focus more on risk assessment, especially when safer alternatives exist.

**Abbreviations**
95%‐CI: 95% confidence interval
AE: Adverse event
AGREE: Classification for adverse events gastrointestinal endoscopy
ASA: American Society of Anesthesiology
ASGE: American Society of Gastroenterology
CI: Confidence interval
cm: centimeter
DBE: Double‐balloon enteroscopy
DY: Diagnostic yield
ERCP: Endoscopic retrograde cholangiopancreaticography
ESGE: European Society of Gastroenterology
FU: Follow‐up
MSE: Motorized spiral enteroscopy
NOS: Newcastle‐Ottawa Scale
OR: Odds ratio
PPPD: Pylorus‐preserving pancreaticoduodenectomy
PSC: Primary sclerosing cholangitis
RCT: Randomized controlled trial
SAE: Serious adverse event
SBE: Single‐balloon enteroscopy
TY: Therapeutic yield

## Conflicts of Interest

Ingo Steinbrück: lecture fees and travel grants from Olympus and Falk Pharma, Siegbert Faiss: lecture fees and consulting fees from Olympus and Ovesco Endoscopy AG, Hendrik Buchholz: no conflicts of interest, Björn Lewerenz: no conflicts of interest, Daniel Fitting: no conflicts of interest, Felix Wiedbrauck: No conflicts of interest, Stephan Hollerbach: no conflicts of interest, Arthur Schmidt: research grants and lecture fees from Ovesco Endoscopy, consulting fees from KLS Martin and lecture fees from Falk Pharma and Olympus Medical, Johannes Wilhelm Rey: no conflicts of interest, Martha M. Kirstein: lecture fees from Olympus Medical and Ovesco, Franz Ludwig Dumoulin: lecture fees and travel grants from Olympus, Fabian Maximilian Wittich: no conflicts of interest, Andreas Wannhoff: research grants from Fujifilm and Ovesco, Jürgen Pohl: no conflicts of interest, Matthias Friesicke: no conflicts of interest, Viktor Rempel: lecture fees from Olympus, Hans‐Peter Allgaier reports lecture fees and travel grants from Olympus. Armin Kuellmer reports lecture fees from Ovesco Endoscopy AG and Falk Pharma and consulting from KLS Martin Group, Tuttlingen, Germany. Thomas Rösch: Research support from Olympus, Fujifilm and Erbe.

## Supporting information


**Figure S1:** AE in the subgroup of peroral MSE (small bowel indications) – Forrest plot of the meta‐analysis.

**Figure S2:** AE in the subgroup of peranal MSE (small bowel indications) – Forrest plot of the meta‐analysis.

**Figure S3:** SAE in the subgroup of peroral MSE (small bowel indications) – Forrest plot of the meta‐analysis.

**Figure S4:** SAE in the subgroup of peranal MSE (small bowel indications) – Forrest plot of the meta‐analysis.

**Figure S5:** AE in the subgroup of peroral DBE/SBE (small bowel indications) – Forrest plot of the meta‐analysis.

**Figure S6:** AE in the subgroup of peranal DBE/SBE (small bowel indications) – Forrest plot of the meta‐analysis.

**Figure S7:** SAE in the subgroup of peroral DBE/SBE (small bowel indications) – Forrest plot of the meta‐analysis.

**Figure S8:** SAE in the subgroup of peranal DBE/SBE (small bowel indications) – Forrest plot of the meta‐analysis.

**Figure S9:** Flow sheet of the German PowerSpiral registry.

**Table S1:** Secondary outcomes in the German PowerSpiral Registry.

**Table S2:** Inclusion and exclusion criteria in the German PowerSpiral Registry.

**Table S3:** Characteristics of the studies of the meta‐analysis.

**Table S4:** Characteristics and performance in the participating centers in the German PowerSpiral Registry.

**Table S5:** Patient and procedural characteristics in the German PowerSpiral Registry.

**Table S6:** Outcomes for Motorized spiral endoscopy with small bowel indication in the German PowerSpiral Registry.

**Table S7:** Outcomes for Motorized spiral endoscopy with ERCP indication in the German PowerSpiral Registry.

**Table S8:** Outcomes for Motorized spiral endoscopy with colonoscopy indication in the German PowerSpiral Registry.

**Table S9:** AE, SAE and incidents in in the German PowerSpiral Registry.

**Table S10:** Cases of Serious Adverse events in the German PowerSpiral Registry (all indications).

**Table S11:** Cases of technical problems/failures of the MSE from the German PowerSpiral Registry (all indications).

**Table S12:** Predictive factors for AE (uni‐/multivariable analysis) in the German PowerSpiral Registry (all indications).

## Data Availability

Research data are not shared.
