# Supplementary material for: Was Motorized Spiral Enteroscopy Too Risky? A Systematic Review and Meta‐Analysis Including German Registry Data
Source: United European Gastroenterol J. 2026 Jan 6;14(1):e70165. doi: 10.1002/ueg2.70165 (PMC12781184; doi:10.1002/ueg2.70165)
Supplement: Supplementary file 2 — Figure S2: AE in the subgroup of peranal MSE (small bowel indications) – Forrest plot of the meta‐analysis. [file UEG2-14-e70165-s010.docx]

**Supplementary Figure 2s: AE in the subgroup of peranal MSE (small bowel indications) – Forrest plot of the meta-analysis**

**
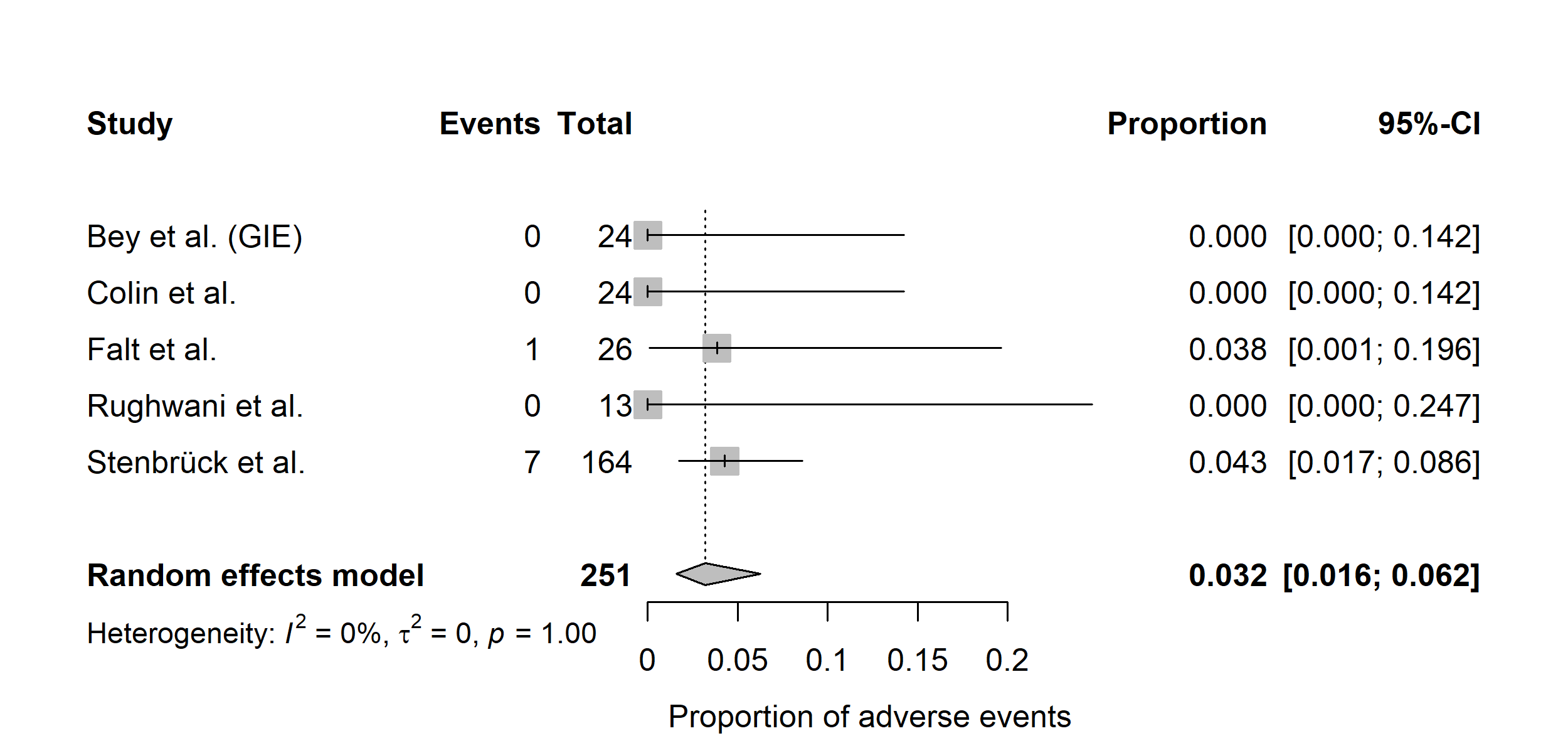
**

AE: Adverse event, MSE: Motorized spiral enteroscopy.
