# Supplementary figures and images for: Was Motorized Spiral Enteroscopy Too Risky? A Systematic Review and Meta‐Analysis Including German Registry Data
Source: United European Gastroenterol J. 2026 Jan 6;14(1):e70165. doi: 10.1002/ueg2.70165 (PMC12781184; doi:10.1002/ueg2.70165)

**Supplementary Figure 9s: Flow sheet of the German PowerSpiral registry**

**
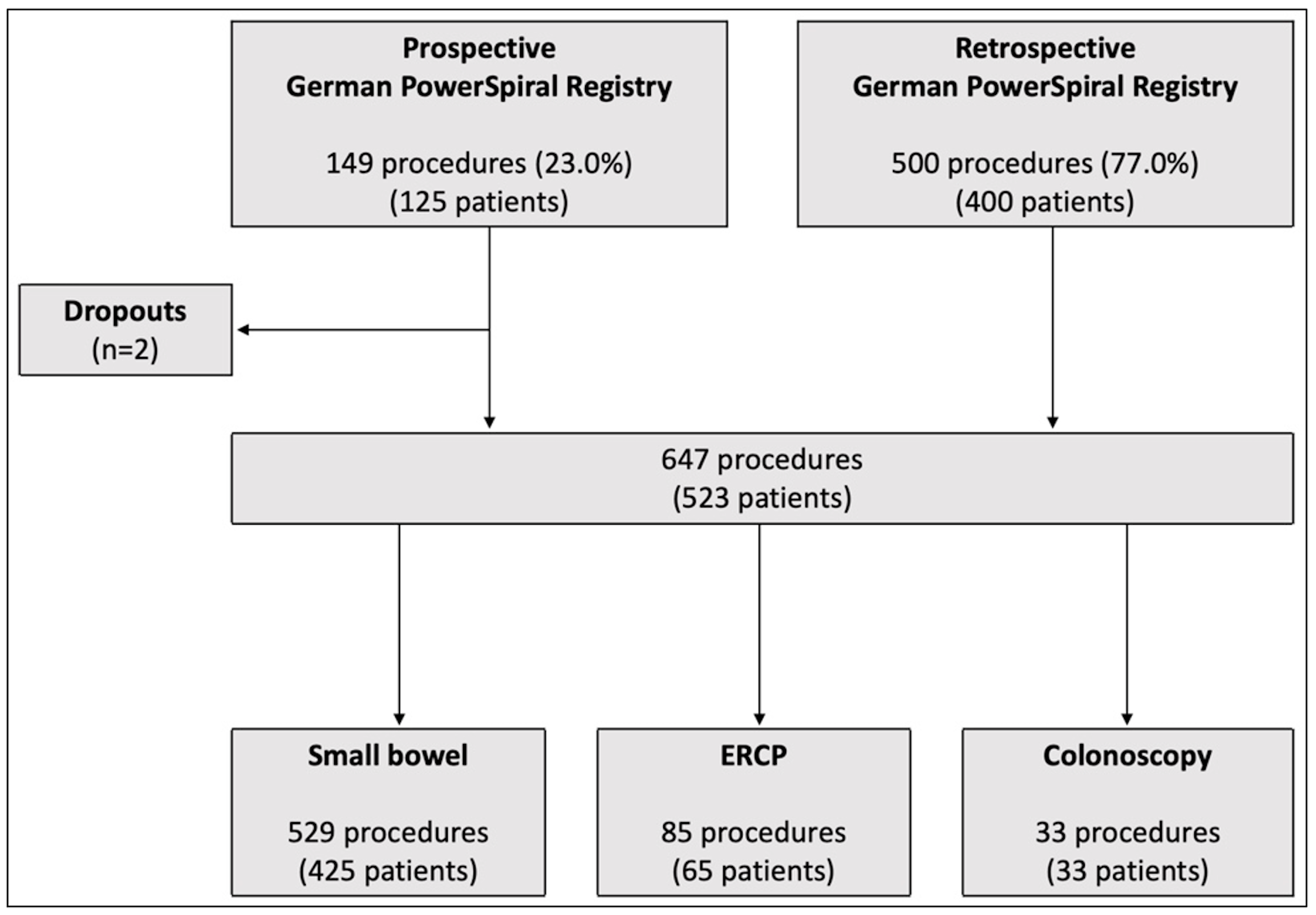
**

Supplement: Supplementary file 9 — Figure S9: Flow sheet of the German PowerSpiral registry. [file UEG2-14-e70165-s018.docx]
