# Supplementary material for: Was Motorized Spiral Enteroscopy Too Risky? A Systematic Review and Meta‐Analysis Including German Registry Data
Source: United European Gastroenterol J. 2026 Jan 6;14(1):e70165. doi: 10.1002/ueg2.70165 (PMC12781184; doi:10.1002/ueg2.70165)
Supplement: Supplementary file 10 — Table S1: Secondary outcomes in the German PowerSpiral Registry. [file UEG2-14-e70165-s019.docx]

**Supplementary Table 1s: Secondary outcomes in the German PowerSpiral Registry**

| **Secondary outcomes** | |
| --- | --- |
| Rate of incidents | unplanned events that do not interfere with completion of the planned procedure or change the plan of care [14], including clinically not relevant mucosal injuries |
| Severity of adverse events | according to [14] |
| Mortality | rate of fatal adverse events [14]/AGREE grade V [15] |
| Type and therapy of adverse events | - |

AGREE: Classification for adverse events gastrointestinal endoscopy.
