# Supplementary material for: Was Motorized Spiral Enteroscopy Too Risky? A Systematic Review and Meta‐Analysis Including German Registry Data
Source: United European Gastroenterol J. 2026 Jan 6;14(1):e70165. doi: 10.1002/ueg2.70165 (PMC12781184; doi:10.1002/ueg2.70165)
Supplement: Supplementary file 11 — Table S2: Inclusion and exclusion criteria in the German PowerSpiral Registry. [file UEG2-14-e70165-s012.docx]

**Supplementary Table 2s: Inclusion and exclusion criteria in the German PowerSpiral Registry**

*(also patients with status post-abdominal surgery were included in both data collections, no exclusion criteria for the retrospective data collection)*

| **Inclusion criteria** |
| --- |
| All MSE that were performed for any indication  (small bowel disease, ERCP in surgically altered anatomy, colonoscopy) |
| **Exclusion criteria (prospective cases only)** |
| Age< 18 years |
| Lack of capability or rejection of giving consent |
| Pregnancy |
| Score of the American Society of Anesthesiologists (ASA) = V |
