# Supplementary material for: Was Motorized Spiral Enteroscopy Too Risky? A Systematic Review and Meta‐Analysis Including German Registry Data
Source: United European Gastroenterol J. 2026 Jan 6;14(1):e70165. doi: 10.1002/ueg2.70165 (PMC12781184; doi:10.1002/ueg2.70165)
Supplement: Supplementary file 12 — Table S3: Characteristics of the studies of the meta‐analysis. [file UEG2-14-e70165-s008.docx]

**Supplementary Table 3s: Characteristics of the studies of the meta-analysis**

| **Authors** | **Year** | **Country** | **Device** | **Design** | **Procedures** | **AE** | **SAE** |
| --- | --- | --- | --- | --- | --- | --- | --- |
| Beyna et al. (Gut) [6] | 2020 | Germany, Belgium | MSE | Prosp. | 140 | 16 | 2 |
| Ramchandani et al.[21] | 2021 | India | MSE | Retrosp. | 83 | 15 | 0 |
| Beyna et al. (GIE) [4] | 2021 | Germany, Belgium | MSE | Prosp. | 54 | 1 | 0 |
| Beyna et al. (Endoscopy) [8] | 2022 | Germany, Belgium, France, Denmark, Finland, Switzerland, Norway | MSE | Prosp. | 337 | 37 | 8 |
| Al-Toma et al. (Endoscopy) [5] | 2022 | Netherlands | MSE | Prosp. | 216 | 15 | 0 |
| Singh et al. (DEN Open) [22] | 2022 | India | MSE | Retrosp. | 75 | 21 | 1 |
| Chan et al. [10] | 2023 | Singapore | MSE | Retrosp. | 39 | 11 | 1 |
| Colin et al. [23] | 2023 | Belgium | MSE | Retrosp. | 75 | 13 | 1 |
| Falt et al. [24] | 2023 | Czech Republic | MSE | Prosp. | 82 | 2 | 2 |
| Rughwani et al. [12] | 2023 | India | MSE/SBE | Prosp. | 48/69 | 3/2 | 0/0 |
| Singh et al. (Cureus) [25] | 2024 | India | MSE | Retrosp. | 20 | 4 | 0 |
| Giordano et al. [26] | 2024 | Spain | MSE | Prosp. | 326 | 35 | 6 |
| Stenbrück et al. | 2025 | Germany | MSE | Pro-/retrosp. | 529 | 34 | 10 |
| Mönkemüller et al. [27] | 2005 | Germany | DBE | Retrosp. | 70 | 0 | 0 |
| May et al. [28] | 2005 | Germany | DBE | Prosp. | 248 | 14 | 0 |
| Heine et al. [29] | 2006 | Netherlands | DBE | Prosp. | 316 | 11 | 1 |
| Mehdizadeh et al. [30] | 2006 | USA | DBE | Prosp. | 232 | 9 | 5 |
| Kaffes et al. [31] | 2006 | Australia | DBE | Prosp. | 62 | 3 | 1 |
| Cazzato et al. [32] | 2007 | Italy | DBE | Prosp. | 118 | 18 | 0 |
| Ohmiya et al. [33] | 2007 | Japan | DBE | Retrosp. | 706 | 12 | 12 |
| Kameda et al. [34] | 2007 | Japan | DBE | Prosp. | 64 | 0 | 0 |
| Zhong et al. [35] | 2007 | China | DBE | Retrosp. | 471 | 4 | 2 |
| Kawamura et al. [36] | 2008 | Japan | SBE | Prosp. | 37 | 1 | 1 |
| Ohtsuka et al. [37] | 2008 | Japan | SBE | n.m. | 48 | 0 | 0 |
| Tsujikawa et al. [38] | 2008 | Japan | SBE | n.m. | 78 | 2 | 1 |
| Ramchandani [39] | 2009 | India | SBE | n.m. | 131 | 8 | 0 |
| Aktas et al. [40] | 2010 | Netherlands | SBE | Prosp. | 166 | 1 | 1 |
| Manner et al. [41] | 2010 | Germany | DBE | Prosp. | 156 | 1 | 0 |
| May et al. [42] | 2010 | Germany | SBE/DBE | Prosp. | 100 | 3 | 0 |
| Frantz et al. [43] | 2010 | USA | SBE | Retrosp. | 38 | 0 | 0 |
| Hegde et al. [44] | 2010 | USA | DBE | Retrosp. | 216 | 2 | 0 |
| Khashab et al. [45] | 2010 | USA | SBE | Retrosp. | 52 | 2 | 1 |
| Pata et al. [46] | 2010 | Turkey | DBE | Retrosp. | 216 | 21 | 8 |
| Godeschalk et al. [47] | 2010 | Netherlands | SBE/DBE | Retrosp. | 151 | 2 | 1 |
| Upchurch et al. [48] | 2010 | USA | SBE | Retrosp. | 172 | 2 | 0 |
| Takano et al. [49] | 2011 | Japan | SBE/DBE | Prosp. | 66 | 2 | 2 |
| Domagk et al. [50] | 2011 | Germany, Netherlands, Norway, | SBE/DBE | Prosp. | 234 | 0 | 0 |
| Jovanovic et al. [51] | 2011 | Germany, Serbia | DBE | Retrosp. | 614 | 5 | 3 |
| Efthymiou et al. [52] | 2012 | Australia | SBE/DBE | Prosp. | 119 | 2 | 0 |
| Gong et al. [53] | 2012 | China | SBE | Retrosp. | 80 | 0 | 0 |
| Byeon et al. [54] | 2012 | USA | DBE | Retrosp. | 203 | 8 | 5 |
| Messer et al. [2] | 2013 | Germany | DBE | Prosp. | 26 | 3 | 0 |
| Manno et al. [55] | 2013 | Italy | SBE | Prosp. | 131 | 0 | 0 |
| Prachayakul et al. [56] | 2013 | Thailand | SBE | Retrosp. | 145 | 10 | 0 |
| Kushnir et al. [57] | 2013 | USA | SBE | Retrosp. | 150 | 1 | 1 |
| Sidhu et al. [58] | 2013 | UK | DBE | Prosp. | 148 | 1 | 1 |
| Baijal et al. [59] | 2014 | India | SBE | Retrosp. | 48 | 2 | 0 |
| Li et al. [60] | 2014 | China | SBE | Prosp. | 365 | 1 | 1 |
| Yamamoto et al. [61] | 2014 | Japan | DBE | Prosp. | 179 | 2 | 0 |
| Lenz et al. [62] | 2014 | Germany, Italy | SBE | Prosp. | 169 | 0 | 0 |
| Rahmi et al. [63] | 2014 | France | DBE | Prosp. | 189 | 12 | 4 |
| Sethi et al. [64] | 2014 | USA | 16 | Retrosp. | 170 | 2 | 1 |
| Choi et al. [65] | 2014 | Korea | DBE | Retrosp. | 218 | 4 | 1 |
| Nakayama et al. [66] | 2014 | Japan | DBE | Retrosp. | 538 | 11 | 9 |
| Feng et al. [67] | 2014 | China | DBE | Prosp. | 20 | 4 | 0 |
| Cangemi et al. [68] | 2015 | USA | DBE | Retrosp. | 215 | 0 | 0 |
| Chen et al. [69] | 2016 | China | DBE | Retrosp. | 729 | 6 | 6 |
| Christian et al. [70] | 2016 | USA | SBE | Retrosp. | 136 | 0 | 0 |
| Lin et al. [71] | 2016 | Taiwan | SBE | Retrosp. | 200 | 3 | 3 |
| Ma et al. [72] | 2016 | China | SBE | Retrosp. | 457 | 0 | 0 |
| Ooka et al. [73] | 2016 | Japan | SBE | Retrosp. | 91 | 3 | 0 |
| Lu et al. [74] | 2017 | China | SBE/DBE | Retrosp. | 173 | 19 | 0 |
| Marques et al. [75] | 2017 | Portugal | SBE | Retrosp. | 197 | 3 | 0 |
| Ching et al. [76] | 2018 | UK | DBE | Prosp. | 215 | 11 | 1 |
| Blanco Velasco et al. [77] | 2020 | Mexico | DBE | Prosp. | 46 | 2 | 0 |
| Liu et al. [78] | 2021 | China | SBE | Prosp. | 220 | 2 | 1 |
| Koh et al. [79] | 2024 | Singapore | SBE/DBE | Retrosp. | 127 | 0 | 0 |

AE: Adverse event, SAE: Serious adverse event, MSE: Motorized spiral enteroscopy, Prosp.: Prospective, Retrosp.: Retrospective, DBE: Double-balloon enteroscopy, SBE: Single-balloon enteroscopy
