# Supplementary material for: Was Motorized Spiral Enteroscopy Too Risky? A Systematic Review and Meta‐Analysis Including German Registry Data
Source: United European Gastroenterol J. 2026 Jan 6;14(1):e70165. doi: 10.1002/ueg2.70165 (PMC12781184; doi:10.1002/ueg2.70165)
Supplement: Supplementary file 13 — Table S4: Characteristics and performance in the participating centers in the German PowerSpiral Registry. [file UEG2-14-e70165-s020.docx]

**Supplementary Table 4s: Characteristics and performance in the participating centers in the German PowerSpiral Registry**

| **Center** | **Number of procedures** | **Retrospective cases** | **Prospective cases** | **Time of recruitment (months)** | **Number of operators** |
| --- | --- | --- | --- | --- | --- |
| **1** | 123 | 123 | - | 38 | 6 |
| **2** | 119 | 98 | 21 | 39 | 2 |
| **3** | 107 | 107 | - | 28 | 3 |
| **4** | 80 | 34 | 46 | 26 | 2 |
| **5** | 62 | 43 | 19 | 36 | 2 |
| **6** | 45 | 16 | 29 | 33 | 2 |
| **7** | 44 | 17 | 27 | 28 | 1 |
| **8** | 32 | 32 | - | 20 | 4 |
| **9** | 22 | 18 | 4 | 30 | 1 |
| **10** | 10 | - | 10 | 10 | 1 |
| **11** | 3 | - | 3 | 13 | 2 |
