# Supplementary material for: Was Motorized Spiral Enteroscopy Too Risky? A Systematic Review and Meta‐Analysis Including German Registry Data
Source: United European Gastroenterol J. 2026 Jan 6;14(1):e70165. doi: 10.1002/ueg2.70165 (PMC12781184; doi:10.1002/ueg2.70165)
Supplement: Supplementary file 14 — Table S5: Patient and procedural characteristics in the German PowerSpiral Registry. [file UEG2-14-e70165-s015.docx]

**Supplementary Table 5s: Patient and procedural characteristics in the German PowerSpiral Registry**

|  | **All**  **647 examinations**  **(523 patients)** | **Indication**  **Small Bowel**  **529 examinations**  **(425 patients)** | **Indication**  **ERCP**  **85 examinations**  **(65 patients)** | **Indication Colonoscopy**  **33 examinations**  **(33 patients)** |
| --- | --- | --- | --- | --- |
| **Patient age** | 65.76 (±15.90; 17-96) | 65.94 (±16.39; 17-96) | 64.28 (±14.57; 20-91) | 66.30 (±11.68; 34-85) |
| **Patient sex**  - male  - female | 310/523 (59.3%)  213/523 (40.7%) | 252/425 (59.3%)  173/425 (40.7%) | 40/65 (61.5%)  25/65 (38.5%) | 18/33 (54.5%)  15/33 (45.5%) |
| **Patient ASA grade**  - I  - II  - III  - IV | 2.39 (±0.87; 1-4)  92/523 (17.6%)  179/523 (34.2%)  207/523 (39.6%)  45/523 (8.6%) | 2.40 (±0.86; 1-4)  72/425 (16.9%)  145/425 (34.1%)  173/425 (40.7%)  35/425 (8.2%) | 2.62 (±0.91; 1-4)  9/65 (13.8%)  17/65 (26.2%)  29/65 (44.6%)  10/65 (15.4%) | 1.82 (±0.68; 1-3)  11/33 (33.3%)  17/33 (51.5%)  5/33 (15.2%)  - |
| **Antiplatelet-/Anticoagulant therapy** | 238/523 (45.5%) | 216/425 (50.8%) | 16/65 (24.6%) | 6/33 (18.2%) |
| **Status post-abdominal surgery** | 205/523 (39.2%) | 131/425 (30.8%) | 65/65 (100%) | 9/33 (27.3%) |
| **Status post-small bowel surgery** | 100/523 (19.1%) | 36/425 (8.5%) | 65/65 (100%) | 1/33 (3.0%) |
| **Indication**  - Bleeding  - Crohn´s disease  - Polyposis syndrome  - Suspected SB disease*  - Celiac disease  - Foreign body removal  - Biliary occlusion  - Biliary stones  - Exchange of drainage  - Biliary fistula  - Incompl. colonoscopy  - Resection right colon | 376/647 (58.1%)  71/647 (11.0%)  33/647 (5.1%)  45/647 (7.0%)  1/647 (0.2%)  3/647 (0.5%)  52/647 (8.0%)  25/647 (3.9%)  7/647 (1.1%)  1/647 (0.2%)  28/647 (4.3%)  5/647 (0.8%) | 376/529 (71.1%)  71/529 (13.4%)  33/529 (6.2%)  45/529 (8.5%)  1/529 (0.2%)  3/529 (0.6%)  -  -  -  -  -  - | -  -  -  -  -  -  52/85 (61.2%)  25/85 (29.4%)  7/85 (8.2%)  1/85 (1.2%)  -  - | -  -  -  -  -  -  -  -  -  -  28/33 (84.9%)  5/33 (15.2%) |
| **Approach**  - Peroral  - Peranal | 449/647 (69.4%)  198/647 (30.6%) | 365/529 (69.0%)  164/529 (31.0%) | 84/85 (98.8%)  1/85 (1.2%) | 0/33 (0%)  33/33 (100%) |
| **Combined approach**  - One session  - Two sessions | 151/647 (23.3%)  54/151 (35.8%)  97/151 (64.2%) | 149/529 (28.2%)  54/149 (36.2%)  95/149 (63.8%) | 2/85 (2.4%)  0/2 (0%)  2/2 (100%) | -  -  - |
| **Endotracheal intubation**  - Peroral  - Peranal | 278/647 (43.0%)  250/449 (55.7%)  28/198 (14.1%) | 230/529 (43.5%)  203/365 (55.6%)  27/164 (16.5%) | 47/85 (55.3%)  47/84 (56.0%)  1/1 (100%) | 1/33 (3.0%)  -  1/33 (3.0%) |
| **Distal cap** | 95/647 (14.7%) | 71/529 (13.4%) | 18/85 (21.2%) | 6/33 (18.2%) |

ERCP: Endoscopic retrograde cholangiopancreaticography, ASA: Amercian Society of Anesthesiologists classification, SB: Small bowel, Incompl.: Incomplete.
