# Supplementary material for: Was Motorized Spiral Enteroscopy Too Risky? A Systematic Review and Meta‐Analysis Including German Registry Data
Source: United European Gastroenterol J. 2026 Jan 6;14(1):e70165. doi: 10.1002/ueg2.70165 (PMC12781184; doi:10.1002/ueg2.70165)
Supplement: Supplementary file 15 — Table S6: Outcomes for Motorized spiral endoscopy with small bowel indication in the German PowerSpiral Registry. [file UEG2-14-e70165-s013.docx]

**Supplementary Table 6s: Outcomes for Motorized spiral endoscopy with small bowel indication in the German PowerSpiral Registry**

| **Indication Small Bowel**  **529 examinations (425 patients)** | |
| --- | --- |
| **Technical success** | 501/529 (94.7%) |
| **Reasons for technical failure**  - no passage of the esophagus  - no passage of the pyloric ring  - no advancement in the duodenum  - no passage of the colon  - no passage of the cecal valve  - defect of the spiral  - AE (respiratory failure) | 7/28 (25.0%)  2/28 (7.1%)  11/28 (39.3%)  2/28 (7.1%)  3/28 (10.7%)  2/28 (7.1%)  1/28 (3.6%) |
| **Complete enteroscopy (all patients)** | 126/425 (29.6%) |
| **Complete enteroscopy (when intended)**  - unidirectional (all unidirectional  examinations)  - bidirectional (all bidirectional  examinations) | Per examination: 165/257 (64.2%) // Per patient: 126/171 (73.7%)  Per examination: 100/372 (26.9%) (peroral: 95 / peranal: 5) // Per patient: 93/347 (26.8%)  Per examination: 65/157 (41.4%) // Per patient: 33/78 (42.3%) |
| **Insertion depth (n=368)**  **-** peroral (n=229)  - peranal (n=139) | 246.97 (±151.87; 0-600) cm  298.01 (±145.11; 0-600) cm  162.88 (±122.89; 0-500) cm |
| **Total procedure time** | 65.26 (±31.47; 0-360) min |
| **Diagnostic yield** | ITT: 322/529 (60.9%) // PP: 320/501 (63.9%) |
| **Diagnosis**  - Angiectasia  - Polyps  - Stenosis  - Crohn´s disease  - Malignoma  - Meckel´s diverticulum  - Ulcus Dieulafoy  - Varices  - Diverticulum  - Inflammation  - Foreign body  - Ulcus  - Various | 101/529 (38.4%)  43/529 (8.1%)  31/529 (5.9%)  16/529 (3.0%)  36/529 (6.8%)  12/529 (2.3%)  11/529 (2.1%)  9/529 (1.7%)  4/529 (0.8%)  4/529 (0.8%)  2/529 (0.4%)  15/529 (2.8%)  42/529 (7.9%) |
| **Therapeutic yield** | ITT: 191/529 (36.1%) // PP: 190/501 (37.9%) |
| **Type of therapy**  **-** APC  - Dilation  - Polypectomy/EMR  - Coagulation  - Foreign body removal  - Injection  - Clipping | 99/191 (51.8%)  20/191 (10.5%)  38/191 (19.9%)  1/191 (0.5%)  2/191 (1.0%)  10/191 (5.2%)  56/191 (29.3%) |

AE: Adverse event, ITT: Intention-to-treat, PP: Per-protocol, APC: Argonplasma coagulation, EMR: Endoscopic mucosal resection.
