# Supplementary material for: Was Motorized Spiral Enteroscopy Too Risky? A Systematic Review and Meta‐Analysis Including German Registry Data
Source: United European Gastroenterol J. 2026 Jan 6;14(1):e70165. doi: 10.1002/ueg2.70165 (PMC12781184; doi:10.1002/ueg2.70165)
Supplement: Supplementary file 16 — Table S7: Outcomes for Motorized spiral endoscopy with ERCP indication in the German PowerSpiral Registry. [file UEG2-14-e70165-s006.docx]

**Supplementary Table 7s: Outcomes for Motorized spiral endoscopy with ERCP indication in the German PowerSpiral Registry**

| **Indication ERCP**  **85 examinations (peroral: 84; peranal: 1)** | |
| --- | --- |
| **Previous surgery**  - BII  - Whipple surgery  - PPPD  - Roux-en-Y without gastrectomy  - Roux-en-Y with partial gastrectomy  - Roux-en-Y with total gastrectomy  - Rou-en-Y with gastric bypass | 12/85 (14.1%)  8/85 (9.4%)  13/85 (15.3%)  33/85 (38.8%)  5/85 (5.9%)  9/85 (10.6%)  5/85 (5.9%) |
| **PTCD Rendez-vous** | 11/85 (12.9%) |
| **Target reached** | 51/85 (60.0%) |
| **Technical success** | 47/85 (55.3%) |
| **Total procedure time** | 69.32 (±34.17; 15-180) min. |
| **Diagnostic yield** | ITT: 43/85 (50.6%) // PP: 38/47 (80.9%) |
| **Diagnosis**  - Biliary stones  - Carcinoma  - Scarring stenosis  - Drainage occlusion  - Stent migration  - PSC | 18/85 (21.2%)  2/85 (2.4%)  18/85 (21.2%)  3/85 (3.5%)  1/85 (1.2%)  1/85 (1.2%) |
| **Therapeutic yield** | ITT: 34/85 (40.0%) // PP: 34/47 (72.3%) |
| **Type of therapy**  - Incision  - Dilation  - Removal of stones  - Drainage  - Removal of drainage | 6/85 (7.1%)  20/85 (23.5%)  13/85 (15.3%)  12/85 (14.1%)  2/85 (2.4%) |

ERCP: Endoscopic retrograde cholangiopancreaticography, BII: Billroth II, PPPD: Pylorus-preserving pancreaticoduodenectomy, PTCD: Percutaneous transhepatic cholangiodrainage.
