# Supplementary material for: Was Motorized Spiral Enteroscopy Too Risky? A Systematic Review and Meta‐Analysis Including German Registry Data
Source: United European Gastroenterol J. 2026 Jan 6;14(1):e70165. doi: 10.1002/ueg2.70165 (PMC12781184; doi:10.1002/ueg2.70165)
Supplement: Supplementary file 17 — Table S8: Outcomes for Motorized spiral endoscopy with colonoscopy indication in the German PowerSpiral Registry. [file UEG2-14-e70165-s001.docx]

**Supplementary Table 8s: Outcomes for Motorized spiral endoscopy with colonoscopy indication in the German PowerSpiral Registry**

| **Indication Colonoscopy**  **33 examinations** | |
| --- | --- |
| **Technical success** | 29/33 (87.9%) |
| **Insertion**  - Ileum  - Cecum  - Ascending colon  - Sigmoid colon | 18/33 (54.5%)  11/33 (33.3%)  2/33 (6.1%)  2/33 (6.1%) |
| **Total procedure time** | 42.53 (±24.03; 5-90) min. |
| **Diagnostic yield** | ITT: 21/33 (63.6%) // PP: 18/29 (62.1%) |
| **Diagnosis**  - Polyp  - Carcinoma  - Angiectasia  - Diverticulosis | 18/33 (54.5%)  3/33 (9.1%)  1/33 (3.0%)  5/33 (15.2%) |
| **Therapeutic yield** | ITT: 19/33 (57.6%) // PP: 17/29 (58.6%) |
| **Type of therapy**  - APC  - Polypectomy/EMR | 1/33 (3.0%)  18/33 (54.5%) |

min.: Minutes, APC: Argonplasma coagulation, EMR: Endoscopic mucosal resection.
