# Supplementary material for: Was Motorized Spiral Enteroscopy Too Risky? A Systematic Review and Meta‐Analysis Including German Registry Data
Source: United European Gastroenterol J. 2026 Jan 6;14(1):e70165. doi: 10.1002/ueg2.70165 (PMC12781184; doi:10.1002/ueg2.70165)
Supplement: Supplementary file 18 — Table S9: AE, SAE and incidents in in the German PowerSpiral Registry. [file UEG2-14-e70165-s014.docx]

**Suppl Table 9s: AE, SAE and incidents in in the German PowerSpiral Registry**

|  | **All**  647 examinations | **Indication**  **Small Bowel**  529 examinations | **Indication**  **ERCP**  85 examinations | **Indication Colonoscopy**  33 examinations |
| --- | --- | --- | --- | --- |
| **All AE**  (=mild/moderate/severe)  - Diagnostic MSE    - Therapeutic MSE | 40 (6.2%)  [4.3-8.2%]*  19/403 (4.7%)  [2.7-7.1%]*  21/244 (8.6%)  [5.2-12.4%]* | 34 (6.4%)  [4.3-8.7%]  17/338 (5.0%) [2.5-7.4%]*  17/191 (8.9%) [4.9-13.3%]* | 5 (5.9%)  [1.2-11.6%]  2/51 (3.9%)  [0.0-10.0%]  3/34 (8.8%)  [0.0-20.0%]* | 1/33 (3.0%)  [0.0-9.1%]  0/14 (0%)  -  1/19 (5.3%)  [0.0-17.6%]* |
| **SAE**  (=moderate/severe)  - Diagnostic MSE  - Therapeutic MSE | 11 (1.7%)  [0.8-2.8%]*  4/403 (1.0%)  [0.2-2.1%]*  7/244 (2.9%)  [0.8-5.1%]* | 10 (1.9%)  [0.8-3.0%]*  4/338 (1.2%)  [0.3-2.6%]*  6/191 (3.1%)  [1.0-5.9%]* | 1 (1.2%)  [0.0-3.6%]*  0/51 (0%)  -  1/34 (2.9%)  [0.0-10.7%]* | 0 (0%)  -  0/14 (0%)  -  0/19 /0%)  - |
| **Severity of AE**  - Mild  - Moderate  - Severe  - Diagnostic MSE  - Therapeutic MSE  - Fatal | 29(4.5%)  4 (0.6%)  7 (1.1%)  3/403 (0.7%)  5/244 (2.0%)  0 (0%) | 24 (4.5%)  3 (0.6%)  7 (1.3%)  3/338 (0.9%)  4/191 (2.1%)  0 (0%) | 4 (4.7%)  1 (1.2%)  0 (0%)  0/51 (0%)  0/34 (0%)  0 (0%) | 1 (3.0%)  0 (0%)  0 (0%)  0/14 (0%)  0/19 (0%)  0 (0%) |
| **Type of AE**  - IPB  - Perforation  - Delayed Perf.  - Resp. failure  - Pancreatitis  - Aspiration  - Abdominal pain  - Nose bleeding  - Fever  - Spiral disconnection  - Spiral retention | 9 (1.4%)  6 (0.9%)  1 (0.2%)  3 (0.5%)  1 (0.2%)  5 (0.8%)  5 (0.8%)  2 (0.3%)  1 (0.2%)  2 (0.3%)  5 (0.8%) | 7 (1.3%)  6 (1.1%)  1 (0.2%)  1 (0.2%)  0 (0%)  5 (0.9%)  4 (0.5%)  2 (0.4%)  1 (0.2%)  2 (0.4%)  5 (0.9%) | 2 (2.4%)  0 (0%)  0 (0%)  1 (1.2%)  1 (1.2%)  0 (0%)  1 (1.2%)  0 (0%)  0 (0%)  0 (0%)  0 (0%) | 0 (0%)  0 (0%)  0 (0%)  1 (3.0%)  0 (0%)  0 (0%)  0 (0%)  0 (0%)  0 (0%)  0 (0%)  0 (0%) |
| **Cause of AE**  - PowerSpiral  - Intervention | 32/40 (80.0%)  8/40 (20.0%) | 28/34 (82.4%)  6/34 (17.6%) | 3/85 (3.5%)  2/85 (2.4%) | 1/33 (3.0%)  0/33 (0%) |
| **Therapy of AE**  - Conservative  - Endoscopic  - Surgery  - Laryngoscopy | 23/40 (57.5%)  10/40 (25.0%)  6/40 (15.0%)  1/40 (2.5%) | 18/34 (52.9%)  9/34 (26.5%)  6/34 (17.6%)  1/34 (2.9%) | 4/5 (80.0%)  1/5 (20.0%)  0/5 (0%)  0/5 (0%) | 1/1 (100%)  0/1 (0%)  0/1 (0%)  0/1 (0%) |
| **Mucosal injuries** | 134 (20.7%) | 121 (22.9%) | 10 (11.8%) | 3 (9.1%) |

ERCP: Endoscopic retrograde cholangiopancreaticography, AE: Adverse event, MSE: Motorized spiral enteroscopy, SAE: Serious adverse event, IPB: Intraprocedural bleeding, Resp.: Respiratory, *: 95%-confidence interval.
