# Supplementary material for: Was Motorized Spiral Enteroscopy Too Risky? A Systematic Review and Meta‐Analysis Including German Registry Data
Source: United European Gastroenterol J. 2026 Jan 6;14(1):e70165. doi: 10.1002/ueg2.70165 (PMC12781184; doi:10.1002/ueg2.70165)
Supplement: Supplementary file 19 — Table S10: Cases of Serious Adverse events in the German PowerSpiral Registry (all indications). [file UEG2-14-e70165-s017.docx]

**Suppl. Table 10s: Cases of Serious Adverse events in the German PowerSpiral Registry (all indications)**

| **Sex** | **Age** | **Approach** | **Type** | **Cause** | **Occurrence** | **Treatment** |
| --- | --- | --- | --- | --- | --- | --- |
| **m** | 19 | Peroral | Jejunal perforation | MSE | <24 hours | Surgery |
| **f** | 79 | Peroral | Jejunal perforation | MSE | Intraprocedural | Surgery |
| **m** | 51 | Peroral | Jejunal perforation | Dilation | Intraprocedural | Surgery |
| **f** | 88 | Peranal | Ileal perforation | MSE | Intraprocedural | Surgery |
| **m** | 75 | Peroral | Jejunal perforation | MSE | <24 hours | Surgery |
| **f** | 87 | Peranal | Ileal edema with abdominal pain | MSE | 24-48 hours | Surgery* |
| **f** | 75 | Peroral | Detachment of the spiral in the upper esophagus | MSE | Intraprocedural | Laryngoscopy |
| **m** | 47 | peroral | Duodenal perforation | MSE | Intraprocedural | Endoscopic |
| **w** | 83 | peroral | Jejunal perforation | Clipping | intraprocedural | Endoscopic |
| **m** | 79 | peroral | Acute pancreatitis | ERCP | <24 hours | Conservative |
| **w** | 74 | peranal | Aspiration | MSE | intraprocedural | Conservative |

m: Male, MSE: Motorized spiral enteroscopy, f: Female, * Laparoscopic exploration, ERCP: Endoscopic retrograde cholangiopancreaticography.
