# Supplementary material for: Was Motorized Spiral Enteroscopy Too Risky? A Systematic Review and Meta‐Analysis Including German Registry Data
Source: United European Gastroenterol J. 2026 Jan 6;14(1):e70165. doi: 10.1002/ueg2.70165 (PMC12781184; doi:10.1002/ueg2.70165)
Supplement: Supplementary file 20 — Table S11: Cases of technical problems/failures of the MSE from the German PowerSpiral Registry (all indications). [file UEG2-14-e70165-s005.docx]

**Supplementary Table 11s: Cases of technical problems/failures of the MSE from the German PowerSpiral Registry (all indications)**

|  | **Approach** | **Type** | **Localization** | **Occurrence** | **Treatment** |
| --- | --- | --- | --- | --- | --- |
| **f 57** | Peroral | Retention of the spiral | Upper esophagus | Withdrawal | Endoscopic removal |
| **f 69** | Peroral | Retention of the spiral | Upper esophagus | Withdrawal | Manual removal |
| **m 81** | Peroral | Retention of the spiral | Upper esophagus | Withdrawal | Endoscopic removal |
| **f 46** | Peroral | Retention of the spiral | Upper esophagus | Withdrawal | Endoscopic removal |
| **f 30** | Peroral | Retention of the spiral | Upper esophagus | Withdrawal | Endoscopic removal |
| **f 75** | Peroral | Detachment of the spiral | Upper esophagus | Withdrawal | Laryngoscopic removal |
| **f 82** | Peroral | Detachment of the spiral | Upper esophagus | Withdrawal | Manual removal |
