# Supplementary material for: Was Motorized Spiral Enteroscopy Too Risky? A Systematic Review and Meta‐Analysis Including German Registry Data
Source: United European Gastroenterol J. 2026 Jan 6;14(1):e70165. doi: 10.1002/ueg2.70165 (PMC12781184; doi:10.1002/ueg2.70165)
Supplement: Supplementary file 21 — Table S12: Predictive factors for AE (uni‐/multivariable analysis) in the German PowerSpiral Registry (all indications). [file UEG2-14-e70165-s009.docx]

**Supplementary Table 12s: Predictive factors for AE (uni-/multivariable analysis) in the German PowerSpiral Registry (all indications)**

| **Variable** | **Univariable analysis** | | **Multivariable analysis** | |
| --- | --- | --- | --- | --- |
| **Impact on AE** | | | | |
|  | **n** | **p** | **Odds ratio [95% CI]** | **p** |
| **Age** | 1-3 | 0.254 |  |  |
| **Sex**  **- male***  **- female** | 18/386 (4.7%)  22/261 (8.4%) | **0.054** | 1.63 (0.84-3.15) | 0.149 |
| **ASA**  **- I***  **- II**  **- III**  **- IV** | 10/116 (8.6%)  14/219 (6.4%)  14/256 (5.5%)  2/56 (3.6%) | 0.453  0.256  0.238 |  |  |
| **Antiplatelet-/Anticoagulant therapy**  **- No***  **- Yes** | 25/355 (7.0%)  15/292 (5.1%) | 0.319 |  |  |
| **Low volume center (1-2 per month)**  **High volume center (>2 per month)*** | 18/218 (5.1%)  22/429 (7.5%) | 0.122 |  |  |
| **Competence level**  **- ≤30 MSE**  **- >30 MSE*** | 21/238 (8.8%)  19/409 (4.6%) | **0.036** | **2.05 (1.07-3.93)** | **0.030** |
| **Approach**  **- Peroral**  **- Peranal*** | 32/449 (7.1%)  8/198 (4.0%) | 0.138 |  |  |
| **Indication**  **- Small Bowel***  **- ERCP**  **- Colonosocopy** | 34/529 (6.4%)  5/85 (5.9%)  1/33 (3.0%) | 0.848  0.445 |  |  |
| **Status post-abdominal surgery**  **- No***  **- Yes** | 22/372 (5.9%)  18/275 (6.5%) | 0.742 |  |  |
| **Status post-small bowel surgery**  **- No***  **- Yes** | 33/501 (6.6%)  7/146 (4.8%) | 0.431 |  |  |
| **Orotracheal intubation**  **- No**  **- Yes*** | 23/369 (6.2%)  17/278 (6.1%) | 0.951 |  |  |
| **Therapeutic procedure**  **- No***  **- Yes** | 19/403 (4.7%)  21/244 (8.6%) | **0.049** | 1.72 (0.89-3.29) | 0.104 |
| **Depth of insertion** |  | 0.313 |  |  |
| **Superficial mucosal injuries**  **- No***  **- Yes** | 22/494 (4.5%)  18/153 (11.8%) | **0.002** | **2.93 (1.52-5.65)** | **0.001** |

AE: Adverse event, 95%-CI: 95%-confidence interval, ASA: Score of the American Society of Anesthesiologists, MSE: Motorized spiral endoscopy, ERCP: Endoscopic retrograde cholangiopancreaticography.
